# Toward Trait-Based Mortality Models for Tropical Forests

**DOI:** 10.1371/journal.pone.0063678

**Published:** 2013-05-13

**Authors:** Mélaine Aubry-Kientz, Bruno Hérault, Charles Ayotte-Trépanier, Christopher Baraloto, Vivien Rossi

**Affiliations:** 1 Université des Antilles et de la Guyane, UMR ‘Ecologie des Forêts de Guyane’, Kourou, France; 2 CIRAD, UMR ‘Ecologie des Forêts de Guyane’, Kourou, France; 3 INRA, UMR ‘Ecologie des Forêts de Guyane’, Kourou, France; Institute of Botany, Czech Academy of Sciences, Czech Republic

## Abstract

Tree mortality in tropical forests is a complex ecological process for which modelling approaches need to be improved to better understand, and then predict, the evolution of tree mortality in response to global change. The mortality model introduced here computes an individual probability of dying for each tree in a community. The mortality model uses the ontogenetic stage of the tree because youngest and oldest trees are more likely to die. Functional traits are integrated as proxies of the ecological strategies of the trees to permit generalization among all species in the community. Data used to parametrize the model were collected at Paracou study site, a tropical rain forest in French Guiana, where 20,408 trees have been censused for 18 years. A Bayesian framework was used to select useful covariates and to estimate the model parameters. This framework was developed to deal with sources of uncertainty, including the complexity of the mortality process itself and the field data, especially historical data for which taxonomic determinations were uncertain. Uncertainty about the functional traits was also considered, to maximize the information they contain. Four functional traits were strong predictors of tree mortality: wood density, maximum height, laminar toughness and stem and branch orientation, which together distinguished the light-demanding, fast-growing trees from slow-growing trees with lower mortality rates. Our modelling approach formalizes a complex ecological problem and offers a relevant mathematical framework for tropical ecologists to process similar uncertain data at the community level.

## Introduction

The dynamics of tree populations in tropical rain forests is a complex ecological process, involving biotic and abiotic interactions between diverse tree species and their environment. Three demographic processes are motors of tree dynamics: recruitment, growth and mortality. Tree recruitment is often defined as recruitment above a minimum DBH (diameter at breast height) [1]. Recruitment depends, on the one hand, on species characteristics related to regeneration (seed mass, dispersal ability 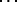) [2]–[5] and, on the other, on diverse environmental variables such as competition for light [6] or soil fertility [7]. Tree growth first depends on the species’ own life-history strategy [8], from fast-growing pioneer species to light-wooded understorey species [9]. In addition, tree growth is mediated by climate [53], environment (light, soil moisture) and competition [11] drivers [12]. Mortality is the least-documented process for diverse reasons. Standing death may occur due to intrinsic senescence [13] or extrinsic agents such as drought or natural enemies. Trees may fall (alone or together) or die standing but may also be broken by wind, rain, or by other falling trees, sometimes causing cascading treefall events [14]. Finally, dominant modes of death may differ in different regions [15]. As a result, tree mortality is a complex phenomenon that hampers the development of robust and predictive forest dynamics models on a large scale.

Mortality is a punctual phenomenon and, moreover, uncommon (rarely exceeding 2 or 3% y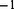 in tropical rain forests all over the world [13], [16]). Like any phenomenon of this kind, difficulty in observing the event renders problematic the understanding of its determinants and, ultimately, its modelling. In this context, mortality modelling has often focused on mortality rates and contrasted these rates between DBH (diameter at breast height) classes [17], [18] or between species groups [1]. However, recent studies suggest that mortality rates are very hard to accurately estimate [19], their value being particularly sensitive to the time between forest censuses [20], [21]. For example, an inaccurate modelling of mortality processes prevents accurate simulation of the spatial variations in above-ground biomass [22]. An alternative strategy is to model the probability of dying at the individual tree level [23], taking species’ ecological strategies into account, in addition to local environmental factors affecting each individual. Such models take advantage of individual tree characteristics, such as past tree growth, neighbouring basal area or current DBH [6], [23], and of the local environment, including competition, climatic variables or soil characteristics. To date, the ecological strategy of each species and the individual vigour of each tree has not been integrated in these approaches. As a starting point, we can suppose that each species has its own ecological strategy, resulting in a unique determinism of the mortality behavior of each species. But the parameterization of such mortality models in mega-diverse communities such as tropical forests poses several problems. First, assuming that it is technically feasible, what interpretation is ecologically meaningful without making the effort to link these behaviours to their biological determinism, i.e. to their functional traits? A second problem is model surparameterization. Indeed, if one wishes to estimate as many model parameters as species, the amount of data to be acquired to obtain robust species estimators is prohibitive. A promising way to solve these two problems simultaneously is to integrate the tree ecological strategy into mortality models through the explicit inclusion of functional traits in the model core, a goal recently achieved for growth models of tropical trees [24], [25].

A central goal of ecology is to understand how variation in the biological properties, i.e. functional traits, of species relates to differences in population dynamics, which, in turn, shape the spatial distribution and temporal fluctuation of communities [26]. Among community ecologists, a consensus is emerging on the existence of different orthogonal axes related to the characteristics of the leaves, wood, seeds and life-history [27], [28]. The leaf economics spectrum opposes inexpensive, short-lived leaves with rapid returns on investments to long-lived leaves with delayed payback times [29]. Wood density is emerging as a core plant functional trait for woody species [30], because it is related to stem construction costs, biomechanics and hydraulic constraints. Seed mass, although not directly related to rates of population dynamics, is an important indicator of the life-history strategy of species, with fast-growing species tending to have small seeds that are easily dispersed [2]. Given that large trait databases on tropical trees are now emerging [30], [31], demonstrating the ability of functional traits to accurately predict mortality behaviour could have important implications for developing robust mortality models in tropical forests.

The paper has three objectives: (i) to present a new community mortality model based on functional traits, (ii) to present an original statistical method used to select the variables and to parameterize the model in a Bayesian framework and (iii) to highlight how species functional traits shape individual tree mortality.

## Materials and Methods

### Data Collection

The study was conducted using data from the Paracou experimental site (5°18′N, 52°55′W), a lowland tropical rain forest near Sinnamary, in French Guiana. The forest is typical of Guianan rain forests, with dominant tree families including Fabaceae, Chrysobalanaceae, Lecythidaceae and Sapotaceae, and with more than 500 woody species attaining 10 cm DBH found at the site. Mean annual precipitation averages 2980 mm (30-y period) with a long dry season from mid-August to mid-November and a short dry season in March [32]. Soils are mostly acrisols, limited in depth by a transformed loamy saprolite (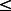 1 m deep), which has a low permeability and leads to lateral drainage during heavy rains [7].

Two data sets are used in the study. The first data set is an inventory of all trees 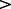
10 cm DBH in 6 natural forest plots of 6.25 ha. Forest inventories were conducted since 1991. Censuses of mortality, recruitment and diameter growth have been conducted every year until 1995 and every 2 years thereafter. We used mortality inventories between 1992 and 2010. The whole data set contained 20,408 individual trees, among which 17,450 were alive in 2010. For each tree in each year, we know the location, DBH, vernacular name and status (dead or alive). The vernacular name is the name used by local treespotters. Botanical determination of the trees was completed in 2012, following extensive inventories with voucher collection and determination at regional and international herbaria. Hence, a large part of the trees that died during the period studied (1992–2010) have no botanical determination, but only a vernacular name.

The second data set is a collection of 15 functional traits of 335 Guianan tree species that occur at the Paracou site. Traits are related to leaf economics, stem economics and life history (Table 1) and are extracted from a large database [24], [28], [31], [33].

**Table 1:** The 15 functional traits used in the study: variable names, units, % of species for which the value of this trait is known in our data set, % of individuals for which the value of this trait is known in our data set, range of the value and results of the Kuo-Mallick (KM) algorithm for variable selection.

Variable	Units	% known	% known	Range		KM
		species	indiv.			
**Leaf economics**						
Foliar 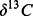 composition ( 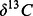 )	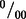	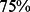	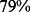	[−36.13	−26.2]	0.044
Foliar 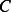 : 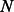 ( 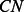 )	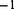 cg g	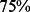	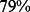	[10.8	46.7]	0.38
Foliar Km ( 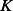 )	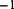 mg g	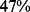	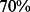	[0.00122	0.223]	0.47
Foliar Nm ( 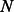 )	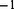 cg g	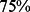	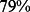	[0.108	0.0451]	0.25
Foliar Pm ( 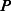 )	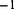 mg g	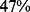	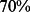	[0.00029	0.00216]	0.34
Leaf tissue density ( 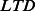 )	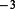 g cm	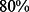	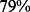	[1.6 10-5	1.4 10-4]	0.31
Laminar total chlorophyll ( 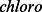 )	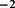 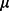 g mm	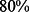	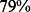	[20.8	149]	0.13
Laminar toughness ( 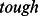 )	N	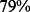	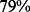	[0.22	11.4]	1
Specific leaf area ( 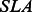 )	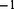 cm 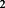 g	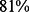	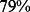	[4.01	37.6]	0.28
**Life history**						
Maximum height ( 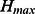 )	m	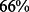	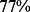	[8	56]	1
Maximum diameter ( 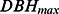 )	mm	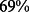	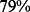	[132	1110]	0.29
Seed mass ( 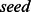 )	g	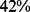	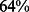	[0.01	20]	0.14
Stem and branch	orthotropic (1)	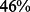	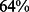	0/1		0.91
orientation ( 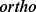 )	plagiotropic (0)					
**Stem economics**						
Trunk wood moisture content		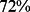	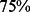	[0.26	1.8]	0.37
( 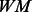 )						
Trunk xylem density ( 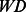 )	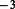 g cm	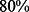	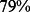	[0.28	0.91]	1

We chose to use a value of 90% as threshold for accepting the trait for inclusion in the final model.

### Addressing Uncertainties in Botanical Determination

The traits data set is complete for 51% of individuals, and our goal was to attribute functional traits to all trees in the database. This is not feasible directly for three cases: (i) the tree species is known but some trait values of the species are not available; (ii) the tree species is not determined at the species level, but only at the family or genus level; (iii) the tree is dead before being identified and only its vernacular name is available. The trees falling in this third case cannot be excluded because they represent 85% of dead trees.

**Figure 1:**
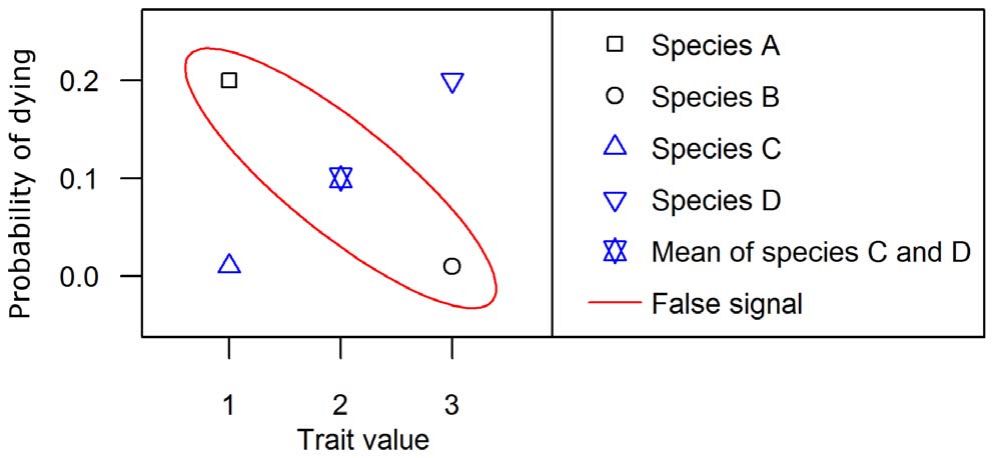
The use of mean traits may produce false signals. Trees of species A and B are botanically well-determined. Trees of species C and D are not, but share the same vernacular name. Using a standard ‘mean-trait’ approach, the mean trait value will be associated with individuals of species C and D, and a false signal may be detected.

**Figure 2:**
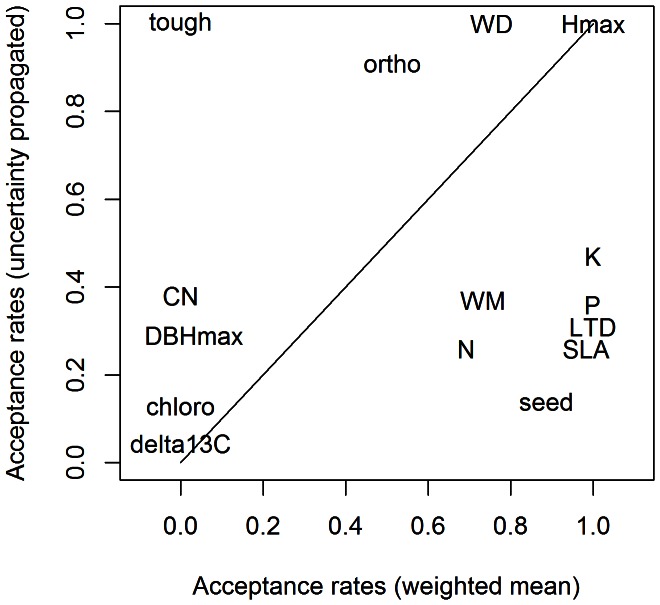
The use of mean traits may produce false signals. Results of the Kuo-Mallick algorithm for parameter selection using weighted means of trait vales are different from what we obtain when we correctly propagate uncertainty.

An intuitive approach is to use a weighted mean of the traits for the missing data. But this approach has some disadvantages. First, some traits are qualitative; attributing a mean value to these traits is not feasible. Most importantly, it could be dangerous to use mean traits. As shown in Figure 1, using means instead of the true values may create an artificial signal in the process we want to model. For instance, if trees of species A and B are well determined but trees of species C and D are not and share the same vernacular name. Using a standard ‘mean-trait’ approach, the mean trait value will be associated with individuals of species C and D, and a false signal may be detected. A final reason for using our method is the uncertainty. If mean traits are used, their values will not be permitted to vary. Then, it will be impossible to propagate the inherent uncertainty of the trait values when observing the uncertainty of the model outputs. Results of the covariates selection using weighted means of the traits and using our method are compared in Figure 2. These results clearly show that using a weighted mean approach would have led to a false trait-based mortality model.

**Figure 3:**
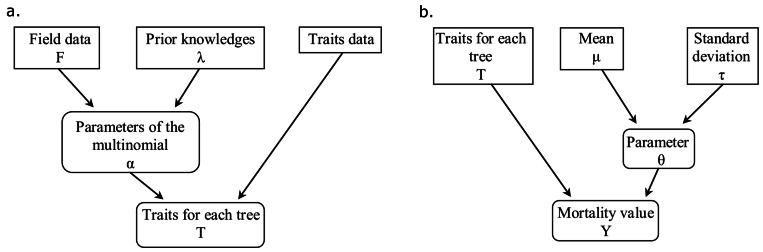
Two steps of model construction. **a:** The functional traits are uncertain variables. The contingency matrix 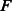 and the prior information 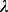 are used in the Dirichlet law to compute the trait variables 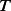 for each tree. **b**: Parameters 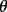 of the model are estimated using a Metropolis-Hastings algorithm with proposal law 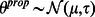. Parameters 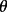 and traits 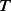 are then used in the final model to compute the mortality measure 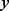 for each tree.

We addressed the cases (i) and (ii) with one relationship model and the case (iii) with a second relationship model (Figure 3). We describe below these relationship models for a single trait 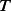, but the same relationship models were used independently for all traits.

The starting point was a set of 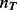 species 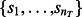 for which we have the associated set of values 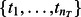 for the trait 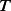. For any tree of a species 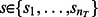, the trait value was set to 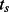. For the other trees, the value of the trait was modelled by a multinomial distribution on the values 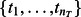 with the associated probabilities computed differently according to the cases (i), (ii) and (iii). The different attribution rules of the trait value are described in the following sections.

#### Attributing trait values to trees of species without known trait values or to trees determined at genus/family levels: cases (i) and (ii)

The studied functional traits are phylogenetically conserved within many families, so taxonomic substitution can be used [34]. This information can be used to fill the gaps of the functional trait data set. For any tree of a species 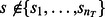 or any tree determined only at the genus/family level, the distribution of trait values was assumed as a multinomial distribution 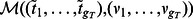. Where 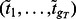 is a subset of 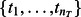 for which the associated species have the same family/genus as the tree; 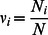, 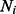 number of trees which have the same family/genus as the tree and have the trait 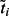; 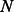 total number of tree which have the same family/genus as the tree.

#### Attributing trait values to trees having only vernacular names: case (iii)

For any tree with a vernacular name 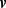, the distribution of trait values was assumed as a multinomial distribution 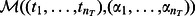. Determination of the probabilities 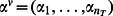 was made using the Bayesian relationship model between the vernacular names and the species determination.

The vernacular name of all trees is known, and therefore can be used as a basis for attributing species. We collected two types of information linking vernacular names to species determination: expert field botanist knowledge linking qualitatively vernacular names to species determination; and field data from the recent inventories, from which we could calculate the frequency with which pairs of vernacular and species names occurred. We then used a Bayesian framework to include these two types of information in the relationship model. The expert knowledge was included as prior information; it informs on which vernacular names are used for which species. For a vernacular name 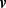, this information can be summed up by a vector 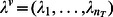 where 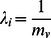 if the link between the species 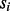 and the vernacular name 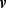 is established by the experts, with 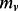 being the number of species linked by the experts with the vernacular name 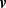; and 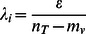 if the link between the species 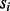 and the vernacular name 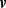 is not established by the experts, with 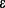 allowing for a small background noise, and 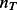 the total number of species in the inventory data.

The field data was included to update the prior information. Trees (alive or dead) for which both the vernacular name and the species name are known allowed us to build for each vernacular name the vector of frequencies belonging to each species. In particular, for the vernacular name 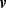, 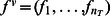 where 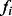 is the number of times a tree with the vernacular name 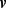 was determinate of species 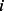.

With these data, 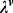 and 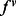, a Multinomial-Dirichlet scheme was used [35]. The expert knowledge, 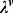, was used as hyperparameters for the prior distribution on 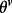: 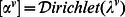. We assumed a multinomial distribution for 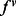 conditionally to 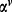. As Multinomial and Dirichlet are conjugate distributions [44], the posterior distribution of 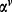 was a Dirichlet distribution 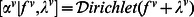.

### Modelling Tree Mortality

**Figure 4:**
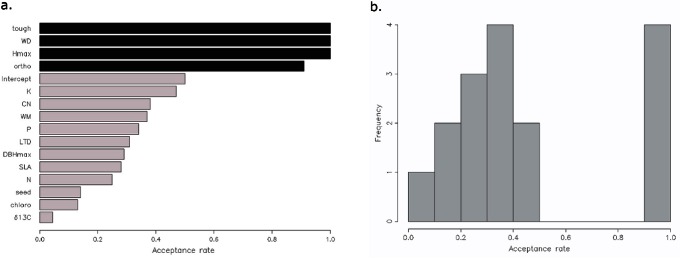
Results of the Kuo-Mallick algorithm for parameter selection. **a:** Mean of the distribution for each variable; variables are included in the final model if the mean value exceeds 90%. Variables included in the final models are: stem and branch orientation (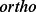), maximum height (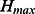), wood density (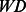) and laminar toughness (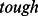). **b:** Histogram of the results of the Kuo-Mallick algorithm for parameter selection. Acceptance rates showed a gap from 0.5 to 0.9, and variables with results above 0.9 were selected for the final model.

We modelled tree mortality with a generalized linear model (GLM) using a logit link function [36]. Tree mortality is a binary variable equal to 0 if the tree remained alive between 1992 and 2010, else equal to 1. The probability of dying for each tree is the logit of a linear combination of a set of covariates: 15 functional trait covariates and 2 ontogenetic covariates 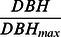 and 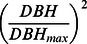. 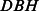 is the diameter of the tree at breast height and 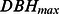 is computed as the 95the percentile of the observed DBH for each species. The ratio 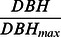 is then used as a proxy of the ontogenetic stage of the tree. As it is well-known that the curve linking mortality probability to ontogenetic stage is U-shaped, we also introduced 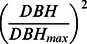 as a covariate. The originality of the model is that the trait covariates are uncertain for some trees and certain for the other trees. We used the method proposed by Kuo-Mallick [37] to select the trait covariates useful to explain mortality. The method consists in multiplying each trait covariate by an indicator. This indicator can be 0 if its covariate is not included in the model or 1 if its covariate is included in the model. We developed a Gibbs algorithm to attribute either 1 or 0 to the indicators and a Metropolis-Hastings within Gibbs algorithm to estimate the coefficients of the covariates. We included a trait covariate in the final model if the expectation of its indicator, given by the Kuo-Mallick method, was between 0.9 and 1 (Figure 4). We chose the threshold 0.9 after considering the histogram of the expectations of the trait covariate indicators (Figure 4). Once the final model was defined, the coefficients of the covariates selected were estimated using the Metropolis-Hastings algorithm. To test the convergence of the chain, we run several chains from diverse initial values and inspect the chains to verify they all converge on the same value after some iterations. The autocorrelation is computed to ensure the chain is mixing adequately. The autocorrelation must decrease to zero when we increase the lag value. The methodology of model building is shown in Figure 3. See Supporting Information S1 and S2 for further information.

All of the algorithms and statistical treatments were implemented with R software [38].

### Model Validation

To validate our mortality model, we split our data into a calibration data set, which contains data between 1992 and 2001, and a validation data set, which contains data between 2001 and 2010. The calibration data set was used to estimate the model parameters. Next, we predicted mortality rates for the validation data set. These rates were computed for different classes of trees binned across the distribution of each functional trait.

## Results

**Figure 5:**
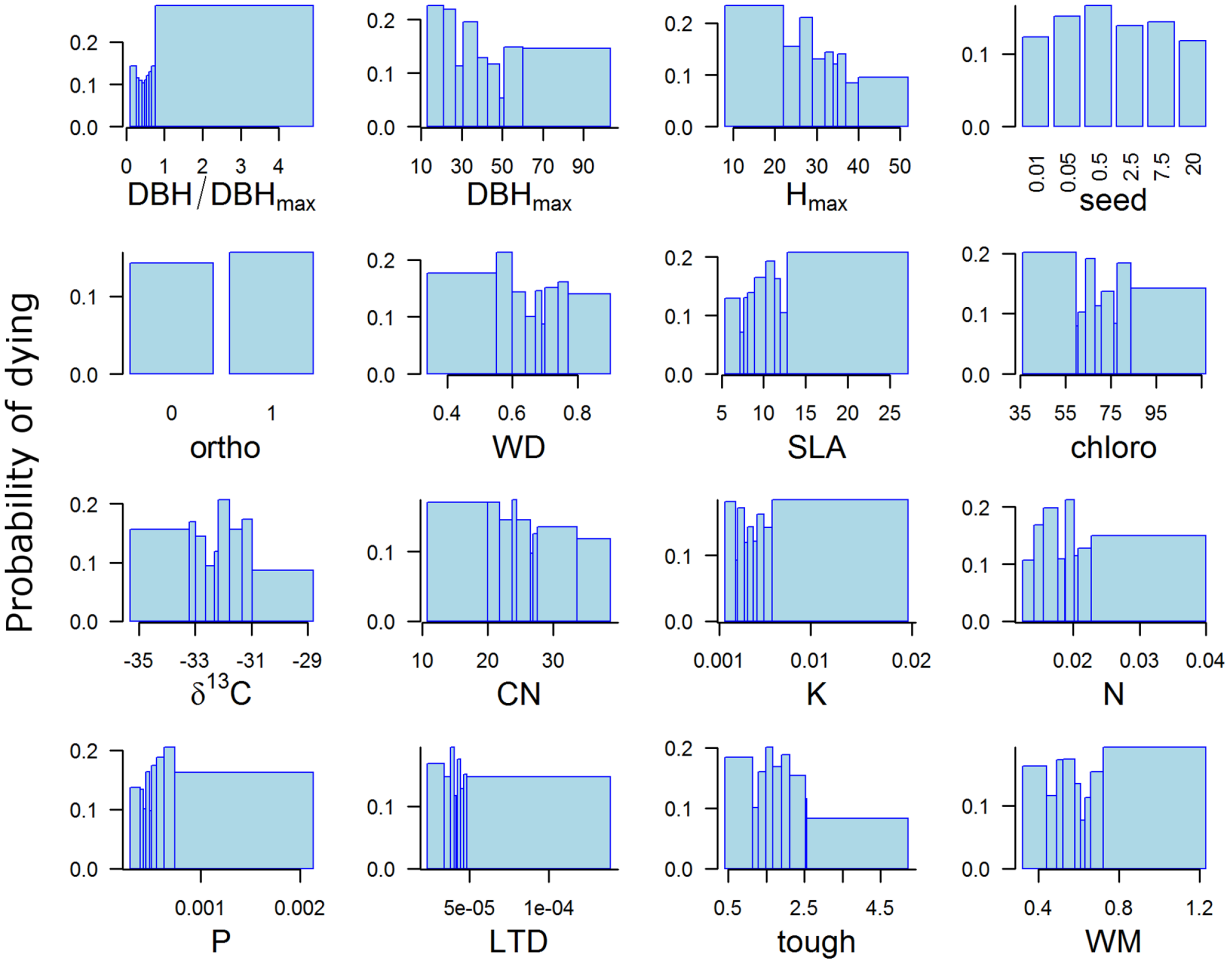
Effects of ontogenetic stage and functional traits on mortality rates. Mortality rates were computed for each class of tree based on distribution deciles and plotted in the form of a histogram. In most cases, trait values are not evenly distributed, and this explains why the size of the bins is not regular. On the top-left histogram, we can see the U-shaped pattern for the ontogenetic variable.

The probability of dying showed a U-shaped pattern, i.e. 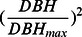 is positively linked and 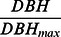 is negatively linked with the mortality probability. This means that the probability of dying first decreased with DBH, was minimum for a DBH ratio close to 0.15 and, then, increased sharply (Figure 5).

**Figure 6:**
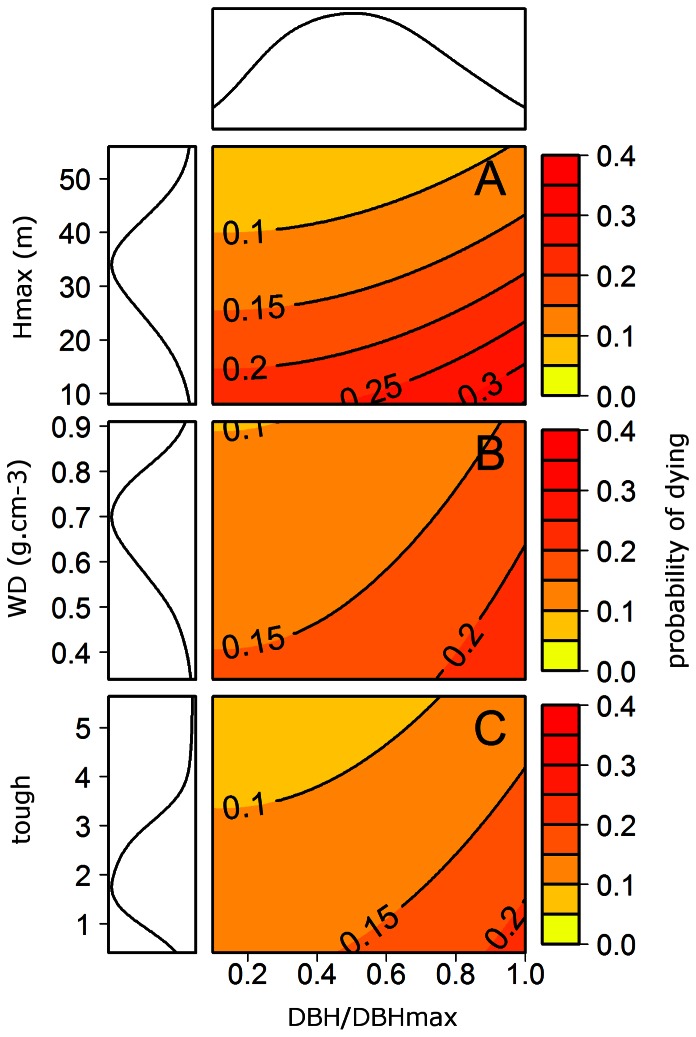
The probability of dying depends on the individual ontogenetic stage and on tree functional traits: the maximum height, Hmax; the wood density, WD; and the laminar toughness, tough. Simulation of the probability of dying versus the ontogenetic stage (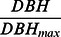) of the tree, with variation of the three continuous functional traits selected in the final model: A the maximum height, B the wood density and C the laminar toughness. Marginal densities are plotted for each trait and for 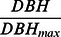, with a bandwidth equal to 10% of the amplitude. Density for laminar toughness (C) shows that maximal values (above 3) are rare; variation of mortality due to this trait is not as strong as the variation due to the maximal height.

The histogram resulting from the Kuo-Mallick selection procedure showed a large break with no values between 0.5 and 0.9 (Figure 4). Predictive functional traits having values above the 0.9 threshold were thus included in the final mortality model (Figure 4): maximum height (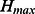), orthotropic orientation (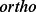), wood density (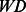) and laminar toughness (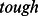). Trees with orthotropic orientation had a lower probability of dying and the other three traits were negatively correlated with the individual probability of dying (Table 2). This means that the probability of dying is even higher when the tree is small, it has orthotropic branches, it has a low density of wood, or it has fragile leaves (Figure 6). Note that functional traits do not modify the shape of the curve linking 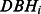 to the dying probability, but functional traits drive the value of this probability in the way that they induce a translation of the curve (Figure 6).

**Table 2:** Results of the Metropolis-Hastings algorithm for parameter estimation.

Variable	Estimates		90% credibility intervals
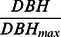	−0	23	[−0.71; 0.26]
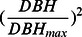	0	78	[0.44; 1.1]
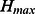	−0	028	[−0.035; −0.022]
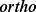	−0	19	[−0.33; −0.045]
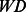	−0	87	[−1.1; −0.55]
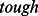	−0	15	[−0.21; −0.078]

Median and 90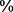 credibility intervals of the posterior distribution for the selected parameters.

**Figure 7:**
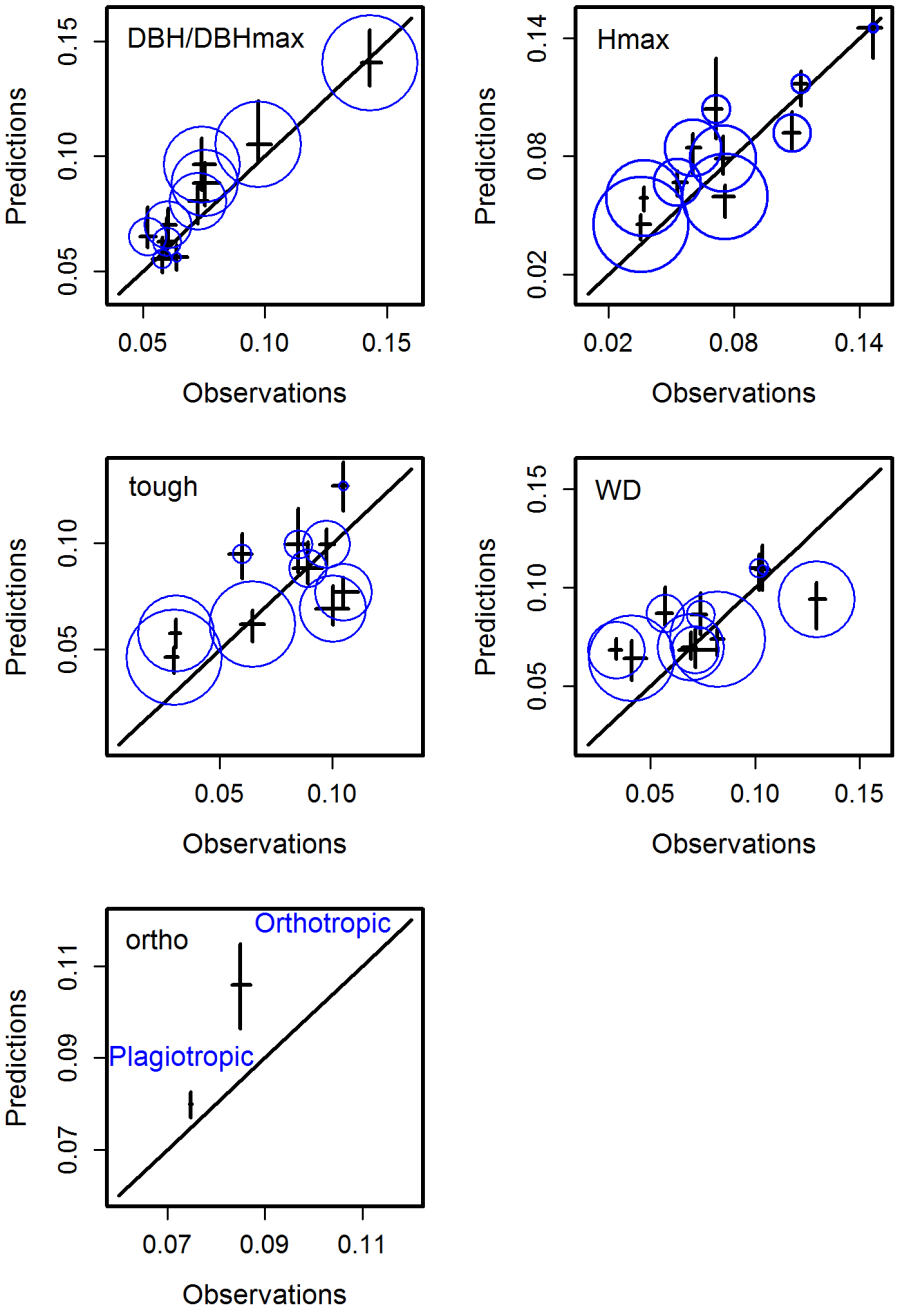
Model validation: the model was calibrated using data from 1992 to 2001 and applied to a validation data set (2001–2010). For each covariate of the mortality model, we binned individuals into ten bins of equal size, corresponding to the deciles of the covariate distribution. Mortality rates were then computed for each bin using the validation data set (2001–2010). Predictions were plotted against observed rates. Moreover, the size of the blue circle is proportional to the value of the median of each bin.

Results of the model validation procedure are presented in Figure 7. For the five covariables, we plotted the predicted rates of tree mortality versus the observed rates of tree mortality. The model, overall, overpredicts the probability of dying (7.6% of observed dead trees versus 8.1% of predicted dead trees) in the prediction data set. The ontogenetic signal has the best fit, especially for high values of 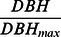. Predictions for 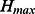 and 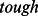 are correct. For 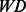, the variability over the observed mortality rates is only slightly represented in the simulated rates. Quite the opposite, for 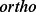, the variability is higher in the predicted rates than in the observation.

## Discussion

This study introduces a new method to design mortality models in tropical forests. Tree ontogenetic stage had an obvious effect on tree mortality, resulting in a somewhat typical U-shaped mortality curve [39], [40]. In other words, young trees and old trees die more frequently [41], probably because of intense competition among the youngest and due to senescence for the oldest. We used functional traits as uncertain covariates of a generalized linear model to predict tree mortality in a tropical rain forest in French Guiana. The results of our study showed that some functional traits are very useful covariates to compute the individual probability of dying for each tree in the forest community. Other parameters, mostly environmental or edaphic, have also been demonstrated to explain the probability of dying [41]–[43]. Our study provides a foundation for coupling both individual characteristics and environmental variables, which we believe will be a promising way to better understand tree mortality and model the consequences of global change on tropical forests.

### Functional Traits

We used an original selection procedure to evaluate which tree functional traits shape the mortality curves. In doing so, we formally incorporated tree functional diversity into forest dynamics modelling without the necessity to build any functional groups. All in all, only four functional traits were robust predictors of the mortality probability. Most of the numerous measured traits related to leaf economics (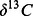, 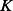
…) were not included in the final model, instead traits related to life history or stem economics were largely selected.

First, wood density was expected to be a good predictor of mortality rates, since [44], [45] reported a robust negative correlation between wood density and tree mortality in Amazonian forests. Moreover, [46] also highlighted the importance of wood density in the trade-off between resource acquisition and investment in survival. In a nutshell, trees with low density are light-demanding trees with rapid bole expansion, leading to a higher mortality probability. High wood density is known to shape resistance to water-stress embolism [47], mechanical breakage or attack by pathogens [30], [48]. This finding is in line with Chao’s hypothesis that species with high mortality rates would create more canopy gaps that, in turn, favor low wood-density species and vice-versa. This means that the mortality regime in tropical forests may be both a cause and an effect of floristic composition [44].


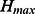
 was predicted to be negatively linked to mortality probability (Figure 5), as reported by [9]. We know that 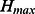 captures a major variation in functional traits found among tropical rain forest tree species, and in combination with light-demand, it provides a rough but straightforward model to understand niche differentiation in tropical forests [49]. 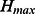 is predicted to be small for light-demanding species with rapid growth and mortality and large for shade-tolerant species with slow growth and mortality [9].

Light-demanding tropical trees are characterized by orthotropic stems and branches, large leaves, and a monolayer leaf arrangement [49]. They are known to realize an efficient height growth through formation of narrow, shallow crowns [49]. Species with orthotropic architecture are therefore expected to be fast-growing and light demanding [50] and, thus, to have higher mortality rates then plagiotropic species. Surprisingly, our model parameters suggest that orthotropic trees have lower mortality rates than plagiotropic trees. We believe this is due to the combined effect of wood density and maximum height, that over-estimated the effect of both traits on mortality. In other words, orthotropic species indeed have higher mortality rates than plagiotropic trees, but the trait model over-predicts this rate for individuals that are small or have a low wood density.

Finally, shade-tolerant plant species have tough leaves because of the high cost of leaf replacement in shade relative to potential carbon gain [51]. Leaf toughness (resistance to fracture per unit fracture area) was the only trait from the leaf economic spectrum to be retained in the mortality model. Leaf toughness was a good candidate because, for some tree saplings, [52] showed that fracture toughness correlated positively with leaf lifespan and survival. Recently, [51] also showed that mortality rates of individuals 1–10 cm in stem diameter were negatively correlated with material toughness and lamina density but were independent of structural toughness and cell-wall fiber content. We extend this finding to adult trees, highlighting the importance of plant-defense traits in shaping individual tree survival.

### Toward New Community Models of Population Dynamics?

In a context of global change, the long-term response of tropical forests to climate change cannot be predicted without using forest simulators or Dynamic Vegetation Models that incorporate both growth and mortality processes. Although tropical forests are known to have very different dynamics regimes, it has been shown that past tree growth is an accurate predictor of tree mortality [44]. The problem with such a result is that it is difficult to use past tree growth per se in a predictive model. Indeed, it only postpones the prediction problem, as it restricts one to predicting events of low growth in the life of a given tree, a goal very hard to achieve across the forest community for at least two reasons. First, most ecological works have focused on the average growth rates [9], as predicting outliers is something extremely complicated in statistical modelling. Second, a tree’s death linked to a decline in vigour appears predictable by its growth pattern before mortality, but tree death caused by disturbance, such as wind, is far from predictable [44]. In this context, we choose to decouple, at first, the growth determinants [24] from the mortality determinants and, then, we hope to find the right ways to combine both processes into a single modelling framework.

The community growth model developed in [24] is based on individual growth computed with the functional traits of trees as covariates. We used the same strategies to build a mortality model, but in our study, we also have to deal with uncertainty of the process of death on the one hand, and with incomplete species information on the other. The strategy of using functional traits does add some uncertainty to the model. The methodology developed in this study handles the uncertainty of the covariates, due both to missing information about the functional traits and to different levels of botanical identification. This kind of incomplete information is common in many tropical forest inventory data sets with high variability in the levels of botanical determination of the trees [53].We used the maximum available information about the trees (species, vernacular name, family/genus) to attribute trait values, under the assumption that functional traits are strongly conserved between species of the same genus and/or the same vernacular name. Although there is some support for this assumption in French Guianan trees [34], the method necessarily involves a loss of some trait information.

Variable selection in nonlinear models remains a complex issue. In our study, the unconventional uncertainties about trait covariates complicated the issue even further. To select the covariates, usual frequentist criteria based on the penalized likelihood, like AIC, BIC, 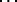, were unusable. Bayesian methods were better adapted because they naturally support different sources of uncertainties in the models. However, using the Bayesian versions of penalized likelihood criteria, such DIC, AICm, 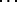, over all combinations of the trait covariates would have required too much computation time. Therefore, we developed a Bayesian algorithm that explores all combinations of trait covariates while calibrating the parameters. There were two possible approaches: Kuo and Mallick [37] or the reversible jump [54]. We chose the Kuo and Mallick approach because it performs better in cases of correlated covariates. Furthermore, its interpretation and implementation are more intuitive because the dimension of the model is not variable, as in the reversible jump approach.

### Conclusion

Our aim in this study was to model tree mortality in a tropical rain forest using functional traits. Considering first the complexity of the mortality process, and second the uncertainty due to the data, the model needed to be developed with particular attention to the methodology. We used a Bayesian framework, on the one hand to use all data about the tree and functional traits at our disposal, and on the other hand, to build the most accurate model using this data. This approach can be generalized to many similar studies about tropical forest dynamics, because more and more data are collected about tree dynamics, but frameworks are missing to correctly process this data. Indeed, ecologists are often limited in their research when working with data from old inventories. Our method should permit increased use of data from old inventories to examine tree mortality. This is particularly interesting for conducting meta-analyses, which are generally based on data with widely varying levels of accuracy.

Tree mortality plays a key-role in the carbon cycle [55], [56] and is intimately linked to forest productivity (e.g. [42]). Global changes, and associated increases in the frequency, duration and/or severity of drought events and heat stress already could have amplified natural tree mortality and potentially will continue to in the future [16], [57], [58], altering tropical forest dynamics and other ecosystem services [59]–[61]. Based on a long-term forest data set, in this study, we developed mortality models suitable for species-rich tropical forest communities, using functional traits as surrogates for taxon-level models. Methods used in this study allow us to model the tree community as a continuum, connecting functional traits to the mortality probability without collapsing species into functional groups.

## Supporting Information

Text S1 **Mortality model equation.**
(PDF)

Text S2 **Algorithms. S2a.** Algorithms for sampling traits value for undetermined trees. **S2b.** Algorithm for estimating parameters in a classical logit model. **S2c.** Algorithm for estimating parameters in a logit model with random covariates. **S2d.** Algorithm for estimating parameters and selecting covariates in a logit model with random covariates.
(PDF)

## References

[1] Alder D, Silva JNM (2000) An empirical cohort model for management of terra firme forests inthe brazilian amazon. Forest Ecology and Management 130: 141–157.

[2] Moles AT, Westoby M (2004) Seedling survival and seed size: a synthesis of the literature. Journal of Ecology 92: 372–383.

[3] Baraloto C, Forget PM, Goldberg DE (2005) Seed mass, seedling size and neotropical tree seedling establishment. Journal of Ecology 93: 1156–1166.

[4] Baraloto C, Forget PM (2007) Seed size, seedling morphology, and response to deep shade and damage in neotropical rain forest trees. American Journal of Botany 94: 901–911. 21636459 10.3732/ajb.94.6.901

[5] Poorter L, Rose S (2005) Light-dependent changes in the relationship between seed mass and seedling traits: a meta-analysis for rain forest tree species. Oecologia 142: 378–387. 15503163 10.1007/s00442-004-1732-y

[6] Gourlet-Fleury S, Cornu G, Jesel S, Dessard H, Jourget JG, et al (2005) Using models to predict recovery and assess tree species vulnerability in logged tropical forests: A case study from French Guiana. Forest Ecology and Management 209: 69–86.

[7] Ferry B, Morneau F, Bontemps JD, Blanc L, Freycon V (2010) Higher treefall rates on slopes and waterlogged soils result in lower stand biomass and productivity in a tropical rain forest. Journal of Ecology 98: 106–116.

[8] Gourlet-Fleury S (1999) Individual-based spatially explicit modelling of forest stands in French Guiana. In: Laumonier Y, King B, Legg C, Renolls K, editors, Data management and modeling using remote sensing and GIS for tropical forest land inventory. Jakarta, Indonesia, 473–490.

[9] Wright SJ, Kitajima K, Kraft NJB, Reich PB, Wright IJ, et al (2010) Functional traits and the growth-mortality trade-off in tropical trees. Ecology 91: 3664–3674. 21302837 10.1890/09-2335.1

[10] Wagner F, Rossi V, Stahl C, Bonal D, Hérault B (2012) Water availability is the main climate driver of neotropical tree growth. Plos One 7: e34074. 22506012 10.1371/journal.pone.0034074 PMC3323616

[11] Uriarte M, Canham CD, Thompson J, Zimmerman JK (2004) A neighborhood analysis of tree growth and survival in a hurricane-driven tropical forest. Ecological Monographs 74: 591–614.

[12] Poorter L (1999) Growth responses of 15 rain-forest tree species to a light gradient: the relative importance of morphological and physiological traits. Functional Ecology 13: 396–410.

[13] Carey EV, Brown S, Gillespie AJR, Lugo AE (1994) Tree mortality in mature lowland tropical moist and tropical lower montane moist forests of Venezuela. Biotropica 26: 255–265.

[14] Jansen PA, Van Der Meer PJ, Bongers F (2008) Spatial contagiousness of canopy disturbance in tropical rain forest: an individual-tree-based test. Ecology 89: 3490–3502. 19137954 10.1890/07-1682.1

[15] Chao KJ, Phillips OL, Monteagudo A, Torres-Lezama A, Martinez RV (2009) How do trees die? Mode of death in northern amazonia. Journal of Vegetation Science 20: 260–268.

[16] Condit R, Hubbell SP, Foster RB (1995) Mortality-rates of 205 neotropical tree and shrub species and the impact of a severe drought. Ecological Monographs 65: 419–439.

[17] Bohlman S, Pacala S (2012) A forest structure model that determines crown layers and partitions growth and mortality rates for landscape-scale applications of tropical forests. Journal of Ecology 100: 508–518.

[18] Condit R, Aguilar S, Hernandez A, Perez R, Lao S, et al (2004) Tropical forest dynamics across a rainfall gradient and the impact of an El Nino dry season. Journal of Tropical Ecology 20: 51–72.

[19] Wagner F, Rutishauser E, Blanc L, Hérault B (2010) Effects of plot size and census interval on descriptors of forest structure and dynamics. Biotropica 42: 664–671.

[20] Sheil D, May RM (1996) Mortality and recruitment rate evaluations in heterogeneous tropical forests. Journal of Ecology 84: 91–100.

[21] Lewis SL, Phillips OL, Sheil D, Vinceti B, Baker TR, et al (2004) Tropical forest tree mortality, recruitment and turnover rates: calculation, interpretation and comparison when census intervals vary. Journal of Ecology 92: 929–944.

[22] Delbart N, Ciais P, Chave J, Viovy N, Malhi Y, et al (2010) Mortality as a key driver of the spatial distribution of aboveground biomass in Amazonian forest: results from a dynamic vegetation model. Biogeosciences 7: 3027–3039.

[23] Phillips PD, de Azevedo CP, Degen B, Thompson IS, Silva JNM, et al (2004) An individualbased spatially explicit simulation model for strategic forest management planning in the eastern Amazon. Ecological Modelling 173: 335–354.

[24] Hérault B, Bachelot B, Poorter L, Rossi V, Bongers F, et al (2011) Functional traits shape ontogenetic growth trajectories of rain forest tree species. Journal of Ecology 99: 1431–1440.

[25] Rüger N, Wirth C, Wright SJ, Condit R (2012) Functional traits explain light and size response of growth rates in tropical tree species. Ecology 93: 2626–2636. 23431593 10.1890/12-0622.1

[26] McGill BJ, Enquist BJ, Weiher E, Westoby M (2006) Rebuilding community ecology from functional traits. Trends in Ecology and Evolution 21: 178–185. 16701083 10.1016/j.tree.2006.02.002

[27] Westoby M (1998) A leaf-height-seed (lhs) plant ecology strategy scheme. Plant and Soil 199: 213–227.

[28] Baraloto C, Paine CET, Poorter L, Beauchêne J, Bonal D, et al (2010) Decoupled leaf and stem economics in rain forest trees. Ecology Letters 13: 1338–1347. 20807232 10.1111/j.1461-0248.2010.01517.x

[29] Wright IJ, Reich PB, Westoby M, Ackerly DD, Baruch Z, et al (2004) The worldwide leaf economics spectrum. Nature 428: 821–827. 15103368 10.1038/nature02403

[30] Chave J, Coomes D, Jansen S, Lewis SL, Swenson NG, et al (2009) Towards a worldwide wood economics spectrum. Ecology Letters 12: 351–366. 19243406 10.1111/j.1461-0248.2009.01285.x

[31] Baraloto C, Paine CET, Patino S, Bonal D, Hérault B, et al (2010) Functional trait variation and sampling strategies in species-rich plant communities. Functional Ecology 24: 208–216.

[32] Wagner F, Hérault B, Stahl C, Bonal D, Rossi V (2011) Modeling water availability for trees in tropical forests. Agricultural and Forest Meteorology 151: 1202–1213.

[33] Hérault B, Ouallet J, Blanc L, Wagner F, Baraloto C (2010) Growth responses of neotropical trees to logging gaps. Journal of Applied Ecology 47: 821–831.

[34] Baraloto C, Hardy OJ, Paine CET, Dexter KG, Cruaud C, et al (2012) Using functional traits and phylogenetic trees to examine the assembly of tropical tree communities. Journal of Ecology 100: 690–701.

[35] Robert CP, Casella G (2004) Monte Carlo statistical methods. Springer, 2nd edition.

[36] McCullagh P, Nelder J (1989) Generalized linear models. Boca Raton: Chapman and Hall, 2nd edition.

[37] Kuo L, Mallick B (1998) Variable selection for regression models. Sankhya SerB : 65–81.

[38] R Development Core Team (2010) R: a language and environment for statistical computing. R Foundation for Statistical Computing, Vienna, Austria. Available: http://www.R-project.org. ISBN 3–900051–07–0.

[39] Muller-Landau HC, Condit RS, Chave J, Thomas SC, Bohlman SA, et al (2006) Testing metabolic ecology theory for allometric scaling of tree size, growth and mortality in tropical forests. Ecology Letters 9: 575–588. 16643303 10.1111/j.1461-0248.2006.00904.x

[40] Coomes DA, Allen RB (2007) Effects of size, competition and altitude on tree growth. Journal of Ecology 95: 1084–1097.

[41] Rüger N, Huth A, Hubbell SP, Condit R (2011) Determinants of mortality across a tropical lowland rainforest community. Oikos 120: 1047–1056.

[42] Stephenson NL, van Mantgem PJ, Bunn AG, Bruner H, Harmon ME, et al (2011) Causes and implications of the correlation between forest productivity and tree mortality rates. Ecological Monographs 81: 527–555.

[43] Toledo JJ, Magnusson WE, Castilho CV, Nascimento HEM (2011) How much variation in tree mortality is predicted by soil and topography in central amazonia? Forest Ecology and Management 262: 331–338.

[44] Chao KJ, Phillips OL, Gloor E, Monteagudo A, Torres-Lezama A, et al (2008) Growth and wood density predict tree mortality in Amazon forests. Journal of Ecology 96: 281–292.

[45] Kraft NJB, Metz MR, Condit RS, Chave J (2010) The relationship between wood density and mortality in a global tropical forest data set. New Phytologist 188: 1124–1136. 21058950 10.1111/j.1469-8137.2010.03444.x

[46] King DA, Davies SJ, Tan S, Noor NSM (2006) The role of wood density and stem support costs in the growth and mortality of tropical trees. Journal of Ecology 94: 670–680.

[47] Jacobsen AL, Ewers FW, Pratt RB, Paddock WA, Davis SD (2005) Do xylem fibers affect vessel cavitation resistance? Plant Physiology 139: 546–556. 16100359 10.1104/pp.104.058404 PMC1203402

[48] Zanne AE, Westoby M, Falster DS, Ackerly DD, Loarie SR, et al (2010) Angiosperm wood structure: Global patterns in vessel anatomy and their relation to wood density and potential conductivity. American Journal of Botany 97: 207–215. 21622380 10.3732/ajb.0900178

[49] Poorter L, Bongers L, Bongers F (2006) Architecture of 54 moist-forest tree species: traits, trade-offs, and functional groups. Ecology 87: 1289–1301. 16761607 10.1890/0012-9658(2006)87[1289:aomtst]2.0.co;2

[50] Poorter L, Bongers F, Sterck FJ, Woll H (2003) Architecture of 53 rain forest tree species differing in adult stature and shade tolerance. Ecology 84: 602–608.

[51] Westbrook JW, Kitajima K, Burleigh JG, Kress WJ, Erickson DL, et al (2011) What makes a leaf tough? patterns of correlated evolution between leaf toughness traits and demographic rates among 197 shade-tolerant woody species in a neotropical forest. American Naturalist 177: 800–811. 10.1086/659963 21597256

[52] Kitajima K, Poorter L (2010) Tissue-level leaf toughness, but not lamina thickness, predicts sapling leaf lifespan and shade tolerance of tropical tree species. New Phytologist 186: 708–721. 20298481 10.1111/j.1469-8137.2010.03212.x

[53] Rejou-Mechain M, Fayolle A, Nasi R, Gourlet-Fleury S, Doucet JL, et al (2011) Detecting largescale diversity patterns in tropical trees: can we trust commercial forest inventories? Forest Ecology and Management 261: 187–194.

[54] Richardson S, Green PJ (1997) On Bayesian analysis of mixtures with an unknown number of components. Journal of the Royal Statistical Society Series B-Methodological 59: 731–758.

[55] Rutishauser E, Wagner F, Hérault B, Nicolini EA, Blanc L (2010) Contrasting above-ground biomass balance in a neotropical rain forest. Journal of Vegetation Science 21: 672–682.

[56] Hérault B, Beauchêne J, Muller F, Wagner F, Baraloto C, et al (2010) Modeling decay rates of dead wood in a neotropical forest. Oecologia 164: 243–251. 20354731 10.1007/s00442-010-1602-8

[57] Phillips OL, van der Heijden G, Lewis SL, Lopez-Gonzalez G, Aragao L, et al (2010) Droughtmortality relationships for tropical forests. New Phytologist 187: 631–646. 20659252 10.1111/j.1469-8137.2010.03359.x

[58] Allen CD, Macalady AK, Chenchouni H, Bachelet D, McDowell N, et al (2010) A global overview of drought and heat-induced tree mortality reveals emerging climate change risks for forests. Forest Ecology and Management 259: 660–684.

[59] Corlett RT (2011) Impacts of warming on tropical lowland rainforests. Trends in Ecology and Evolution 26: 606–613. 21803440 10.1016/j.tree.2011.06.015

[60] Lewis SL (2006) Tropical forests and the changing earth system. Philosophical Transactions of the Royal Society B-Biological Sciences 361: 195–210. 10.1098/rstb.2005.1711 PMC1626535 16553317

[61] Condit R, Hubbell SP, Foster RB (1996) Assessing the response of plant functional types to climatic change in tropical forests. Journal of Vegetation Science 7: 405–416.

