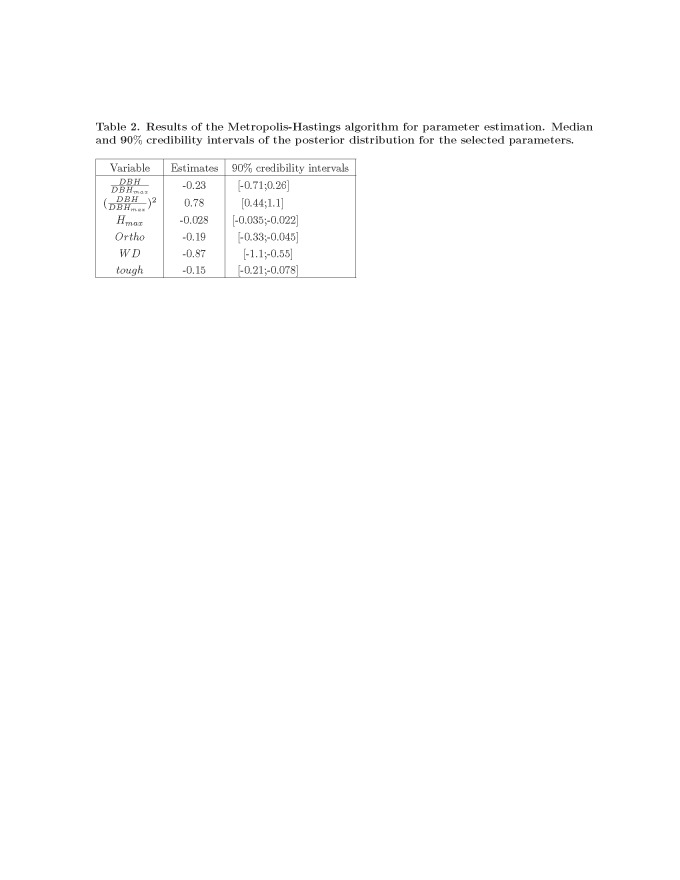# Correction: Toward Trait-Based Mortality Models for Tropical Forests

**DOI:** 10.1371/annotation/af025e30-f31f-4300-be8b-4655484ed09e

**Published:** 2013-11-12

**Authors:** Mélaine Aubry-Kientz, Bruno Hérault, Charles Ayotte-Trépanier, Christopher Baraloto, Vivien Rossi

Formatting errors occurred in Table 1 and Table 2 which have affected the understanding of the data.

Please see the corrected Table 1 here: 

**Figure:**
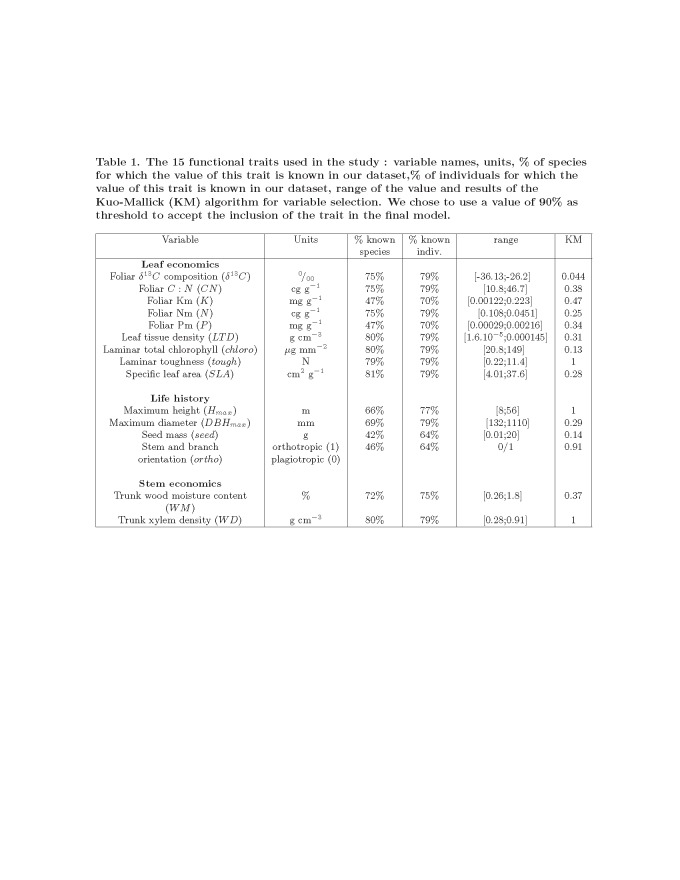


Please see the corrected Table 2 here: 

**Figure:**