# Supplementary material for: Toward Trait-Based Mortality Models for Tropical Forests
Source: PLoS One. 2013 May 13;8(5):e63678. doi: 10.1371/journal.pone.0063678 (PMC3652824; doi:10.1371/journal.pone.0063678)
Supplement: Text S1 — Mortality model equation. (PDF) [file pone.0063678.s001.pdf]

Supporting Information, text S1
Mortality model equation

Tree mortality is a binary variable y_i equal to 0 if tree i is alive, else equal to 1. The mortality model is a generalized linear model, which computes a tree-individual probability of dying. The probability of dying p_i for tree i is calculated with a logit function:

$$p_i = logit^{-1}(LC_i) = \frac{LC_i}{1 + \exp(LC_i)}$$

where LC_i is a linear combination of the covariates associated with the tree i. The covariates are 15 functional traits of tree i and two ontogenetic variables $\left(\frac{DBH_i}{DBH_{max,i}} \text{ and } (\frac{DBH_i}{DBH_{max,i}})^2\right)$, included to estimate the ontogenetic stage of tree i [1]:

$$LC_i = \theta_0 + \theta_1 \frac{DBH_i}{DBH_{max,i}} + \theta_2 \left(\frac{DBH_i}{DBH_{max,i}}\right)^2 + \theta_3 \delta^{13}C_i + \theta_4 CN_i + ...$$

where $\theta = \theta_0, \theta_1, \theta_2, \cdots \theta_m$ is a vector of parameters to be estimated. The mortality value y_i for tree i is then $y_i \sim Bern(p_i)$.

References

1. Hérault B, Bachelot B, Poorter L, Rossi V, Bongers F, et al. (2011) Functional traits shape ontogenetic growth trajectories of rain forest tree species. Journal of Ecology 99: 1431-1440.
