# Supplementary material for: Toward Trait-Based Mortality Models for Tropical Forests
Source: PLoS One. 2013 May 13;8(5):e63678. doi: 10.1371/journal.pone.0063678 (PMC3652824; doi:10.1371/journal.pone.0063678)
Supplement: Text S2 — Algorithms. S2a. Algorithms for sampling traits value for undetermined trees. S2b. Algorithm for estimating parameters in a classical logit model. S2c. Algorithm for estimating parameters in a logit model with random covariates. S2d. Algorithm for estimating parameters and selecting covariates in a logit model with random covariates. (PDF) [file pone.0063678.s002.pdf]

Supporting Information, text S2
Algorithms

S2a. Algorithms for sampling traits value for undetermined trees

We sampled the trait values using different algorithms, according to the level of determination of the trees. (i) If the tree species is known but some trait values of the species are not attributed, we used the algorithm 1 to sample the missing trait values.

for *each determined tree, with some unattributed trait values* **do**
  for *each trait* T *with unattributed value for the tree* **do**
    - let n_T the number of species in the same genus with the value of trait T attributed: $t_1, \ldots, t_{n_T}$;
    - for $i = 1, \ldots, n_T$, let $\nu_i = \frac{N_i}{N}$ with N_i the number of trees in the genus with the trait value t_i and N the number of trees in the genus ;
    - sample $\tilde{t} \sim \mathcal{M}\big((t_1, \ldots, t_{n_T}), (\nu_1, \ldots, \nu_{n_T})\big)$, the value for the trait T for the tree;
  end
end

Algorithm 1: Multinomial algorithm for sampling traits values - case (i)

For the trees determined at the genus/family level we used the algorithm 2.

for *each tree determined at the genus/family level* **do**
  for *each traits* T **do**
    - let n_T the number of species in the same genus/family with the value of trait T attributed: $t_1, \ldots, t_{n_T}$;
    - for $i = 1, \ldots, n_T$, let $\nu_i = \frac{N_i}{N}$ with N_i the number of trees in the genus/family with the trait value t_i and N the number of trees in the genus ;
    - sample $\tilde{t} \sim \mathcal{M}\big((t_1, \ldots, t_{n_T}), (\nu_1, \ldots, \nu_{n_T})\big)$, the value for the trait T for the tree;
  end
end

Algorithm 2: Multinomial algorithm for sampling traits values - case (*ii*)

For the trees with only a vernacular name we used the algorithm 3.

for *each tree with only a vernacular name* **do**
  let v the vernacular name of the tree;
  for *each traits T* **do**
    - let n_T the number of species with the value of trait T attributed: $t_1, \ldots, t_{n_T}$;
    - let $f^v = (f_1, \ldots, f_{n_T})$ the number of trees with vernacular name v which were determined among these species;
    - let m_v the number of species among the n_T for which the expert confirmed a link with the vernacular name v;
    - let $\lambda^v = (\lambda_1, \ldots, \lambda_{n_T})$ with $\lambda_i = \begin{cases} \frac{1}{m_v} & \text{if the link is confirmed} \\ \frac{\varepsilon}{n_T - m_v} & \text{otherwise} \end{cases}$;
    - sample $(\tilde{\alpha}_1, \ldots, \tilde{\alpha}_{n_T}) \sim \mathcal{D}ir(f^v + \lambda^v)$;
    - sample $\tilde{t} \sim \mathcal{M}\big((t_1, \ldots, t_{n_T}), (\tilde{\alpha}_1, \ldots, \tilde{\alpha}_{n_T})\big)$, the value for the trait T for the tree;
  end
end

Algorithm 3: Multinomial algorithm for sampling traits values - case (iii)

Practically, to implement the algorithm 3 on our case study we used $\varepsilon = 0.1$.

S2b. Algorithm for estimating parameters in a classical logit model

First, let us assume that the covariates (the 15 functional traits) are completely known for all the trees. The model is :

$$y_i \sim Bern\left(logit(\theta_0 + \theta_1 x_{i1} + \theta_2 x_{i2} + \cdots + \theta_m x_{im})\right).$$

The likelihood function is :

$$\mathcal{L}(Y|X, \theta) = \prod_{i=1}^{n} \frac{exp((\theta_0 + \theta_1 x_{i1} + ... + \theta_m x_{im}) y_i)}{1 + exp(\theta_0 + \theta_1 x_{i1} + ... + \theta_m x_{im})}$$

where

- $Y = y_1, y_2, ..., y_n$ is the vector of mortality values for all trees,
- $X = x_{i1}, x_{i2}, ..., x_{im}$ is the vector of covariates for tree i (ontogenetic variables and traits),
- $\theta = \theta_1, \theta_2, ..., \theta_m$ is the vector of parameters of the model.

The estimation of parameters θ in the Bayesian framework consists in estimating the posterior distribution of the parameters $\pi_{\theta|Y,X}$. From the Bayesian formula we have $\pi_{\theta|Y,X}(\theta) \propto \mathcal{L}(Y|X,\theta)\pi^0_\theta(\theta)$ where π^0_θ is the prior distribution set by the user on θ. To estimate the posterior distribution $\pi_{\theta|Y,X}$ we can use a Monte Carlo Markov Chain algorithm, like Metropolis-Hastings (cf. algorithm 4). Such algorithm only requires a proposal distribution π^{prop} to sample possible values for θ and this values are kept or not in the chain according to their likelihoods.

Initialisation: $t = 1$, $\theta^t \sim \pi^0_\theta$;

repeat

 Sample $\theta^* \sim \pi^{prop}$;

 Sample $u \sim Unif[0,1]$;

 Compute $\rho(\theta^t, \theta^*) = \dfrac{\mathcal{L}(Y|X,\theta^*)\pi^0_\theta(\theta^*)}{\mathcal{L}(Y|X,\theta^t)\pi^0_\theta(\theta^t)} \dfrac{\pi^{prop}(\theta^t)}{\pi^{prop}(\theta^*)}$;

 if $u < \rho(\theta^t, \theta^*)$ **then**

  $\theta^{t+1} = \theta^*$

 else

  $\theta^{t+1} = \theta^t$

 end

 t=t+1

until *the chain* $(\theta^t)_{t=1,...}$ *reaches its stationary state*;

Algorithm 4: Metropolis-Hastings

S2c. Algorithm for estimating parameters in a logit model with random covariates

However, in our case study, the covariates are not completely known for all the trees. In the paper we built models of knowledge for the traits, thus, the covariates become random for some trees. Let us note

$\pi_{X|i}$ the distribution of the covariate values for the tree i as defined by the algorithm described in the section . For a tree i, for which all traits values are known, $\pi_{X|i}$ is a Dirac measure on its traits values i.e. sampling from $\pi_{X|i}$ always return the traits values of the tree.

Thanks to the Bayesian framework, we could take the randomness on X into account while estimating θ. We must add a Monte-Carlo step into the Metropolis-Hastings algorithm. Practically, we run the Metropolis-Algorithm and new values for the covariates are sampled at each iteration (cf. algorithm 5).

Initialisation: $t = 1$, $\theta^t \sim \pi^0_\theta$;

repeat

  Sample covariate values for each individual i: $\tilde{X}_i \sim \pi_{X,i}$;

  Sample $\theta^* \sim \pi^{prop}$;

  Sample $u \sim Unif[0,1]$;

  Compute $\rho(\theta^t, \theta^*) = \dfrac{\mathcal{L}(Y|\tilde{X}, \theta^*)\pi^0_\theta(\theta^*)}{\mathcal{L}(Y|\tilde{X}, \theta^t)\pi^0_\theta(\theta^t)} \dfrac{\pi^{prop}(\theta^t)}{\pi^{prop}(\theta^*)}$;

  if $u < \rho(\theta^t, \theta^*)$ **then**

    $\theta^{t+1} = \theta^*$

  else

    $\theta^{t+1} = \theta^t$

  end

  t=t+1

until *the chain* $(\theta^t)_{t=1,...}$ *reaches its stationary state*;

Algorithm 5: Monte Carlo within Metropolis-Hastings for a model with random explicative variables

S2d. Algorithm for estimating parameters and selecting covariates in a logit model with random covariates

The last methodological point of the paper was to select useful covariates to explain the mortality while taking their randomness into account. We used a method proposed by [1] which consists in adding an indicator for each covariate :

$$y_i \sim Bern\left(logit(\theta_0 + \theta_1 I_1 x_{i1} + \theta_2 I_2 x_{i2} + \cdots + \theta_m I_m x_{im})\right).$$

These indicators, $I_1, \ldots, I_m$, are considered as unknown parameters. This parameters have to be estimates in addition to θ. We added some steps, inspired from [1], in the algorithm 5 to estimate the posterior distribution of the indicators while taking into account the randomness of the covariates (cf. algorithm [1]). The idea of that method is that if the posterior distribution of the indicator I_j has its mode close to 1, then the covariate j must be kept in the model, otherwise the covariate j must be discarded.

Initialisation: $t = 1$, $\theta^t \sim \pi^0_\theta$, $I_j^{(t)} \sim \mathcal{B}er(0.5)$ for $j = 1, \ldots, m$;

repeat

Sample covariates values for each individual i: $\tilde{X}_i \sim \pi_{X,i}$;

for *each covariate j (selected in a random order)* **do**

Update θ_j

$\theta^* = \theta^t$;

Sample $\theta^*_j \sim \pi^{prop}_{\theta_j|I^{(t)}}$;

Sample $u \sim Unif[0, 1]$;

Compute $\rho(\theta^t, \theta^*) = \dfrac{\mathcal{L}(Y|\tilde{X}, \theta^*)\pi^0_\theta(\theta^*)}{\mathcal{L}(Y|\tilde{X}, \theta^t)\pi^0_\theta(\theta^t)} \dfrac{\pi^{prop}(\theta^t)}{\pi^{prop}(\theta^*)}$;

if $u < \rho(\theta^t, \theta^*)$ **then**

$\theta^t_j = \theta^*_j$

end

Update I_j

Compute $\rho = \dfrac{1}{1+\frac{\mathcal{L}(Y|\tilde{X},\theta^t,I_j=0,I^t_{-j})}{\mathcal{L}(Y|\tilde{X},\theta^t,I_j=1,I^t_{-j})}}$;

Sample $I_j^{(t+1)} \sim Bern(\rho)$;

end

$\theta^{t+1} = \theta^t$;

$t = t + 1$;

until *the chain* $(\theta^t)_{t=1,\ldots}$ *reach its stationary state*;

Algorithm 6: Kuo-Mallick method: Gibbs algorithm to determine the indicators I

Practically, to implement the algorithm [1] on our case study we used :

- a low informative prior distribution on the parameters, $\pi^0_\theta = \mathcal{N}(0, 10^6)$.

- a normal proposal distribution, $\pi^{prop} = \mathcal{N}(\hat{\theta}, \tau^2 I^{-1}(\hat{\theta}|Y))$, where:

  - $\tau = 2$, this parameter is chosen to change the scale of the algorithm steps and have a good acceptance rate.

  - $\hat{\theta}$ is the maximum likelihood estimate,

  - $I^{-1}(\theta|Y)$ is the Fisher matrix information:
    $I(\theta|Y) = \mathbb{E}\left[-\frac{\partial^2 \ell(\theta|Y)}{\partial\theta\partial\theta^t}\right]$, with $\ell(Y|X,\theta) = log(\mathcal{L}(Y|X,\theta))$.

This proposal distribution was used for a similar problem in one dimension by A. Altaleb and C. Robert [2].

References

1. Kuo L, Mallick B (1998) Variable selection for regression models. Sankhya SerB : 65-81.

2. Altaleb A, Robert CP (2001) Analyse bayésienne du modèle logit: algorithme par tranches ou metropolis-hastings? Revue de statistique Appliquée 49: 53-70.
